# A Dual-Reporter Fluorescence–Luminescence Assay for Drug Screening in Promastigote and Intracellular Amastigote Stages of *Leishmania*

**DOI:** 10.3390/molecules31122041

**Published:** 2026-06-11

**Authors:** Sarah D’Alessandro, Silvia Parapini, Estefanía Calvo-Alvarez, Federica Perego, Gaia Mazza, Abirla Murugan, Nicoletta Basilico

**Affiliations:** 1 Department of Pharmacological and Biomolecular Sciences “Rodolfo Paoletti”, University of Milan, 20133 Milan, Italy; sarah.dalessandro@unimi.it (S.D.); estefania.calvo@unimi.it (E.C.-A.); 2 Department of Biomedical Sciences for Health, University of Milan, 20133 Milan, Italy; 3 Department of Biomedical, Surgical and Dental Sciences, University of Milan, 20122 Milan, Italy; federica.perego@unimi.it (F.P.); gaia.mazza@unimi.it (G.M.); abirla.murugan@unimi.it (A.M.); nicoletta.basilico@unimi.it (N.B.)

**Keywords:** *Leishmania infantum*, drug screening, dual-reporter assay, bioluminescence, fluorescence, intramacrophage amastigotes

## Abstract

Leishmaniasis is a Neglected Tropical Disease caused by protozoa of the *Leishmania* genus. No human vaccine is available, and current treatments are limited by toxicity and drug resistance. Thus, the identification of new active molecules remains an urgent priority. However, drug discovery is severely hampered by the lack of fast, robust, and high-throughput screening assays, particularly for the clinically relevant intracellular amastigote stage. Here, a transgenic *Leishmania infantum* line stably co-expressing the bioluminescent marker PpyRE9H fused to the fluorescent reporter tdTomato was generated and used to develop a novel dual-reporter microplate-based platform to identify compounds active against promastigotes or intracellular parasite stages. The engineered strain exhibited comparable growth and infectivity to the wild-type strain, validating its suitability for drug screening assays. The activity of reference drugs was evaluated, and results were compared to those obtained using standard methods, such as MTT assay for promastigotes and Giemsa staining with microscopic quantification for amastigotes. Comparable IC_50_ values were obtained using fluorescent/bioluminescent assays and conventional methods. These proposed assays provide a sensitive and reproducible alternative to conventional methods. The microplate format enables higher throughput screening, while the dual bioluminescence and fluorescence readouts enable cross-validation of results and reduce the risk of compound-specific signal interference.

## 1. Introduction

Leishmaniases are vector-borne Neglected Tropical Diseases caused by protozoan parasites of the genus *Leishmania* and are transmitted through the bite of infected phlebotomine sandflies. The disease is endemic in 98 countries, predominantly in low-income regions of the tropics, and its epidemiology is strongly shaped by geographic, ecological, and vector-related factors. Globally, leishmaniasis is responsible for more than 700,000 new cases and approximately 20,000 deaths each year [1]. Ongoing climate change is contributing to the expansion of sandfly habitats, with increasing reports of leishmaniasis in areas previously considered non-endemic, including several regions of Europe [2,3].

The clinical manifestation and severity of leishmaniasis are determined by a combination of parasite, host, and vector factors, all of which interact within a complex immunological network. During transmission, flagellated promastigotes are inoculated into the mammalian host by the bite of an infected female sandfly. Once inside the host, promastigotes are rapidly internalized by macrophages, where they differentiate into amastigotes and proliferate. The parasite life cycle is completed when a sandfly ingests infected cells during a subsequent blood meal [4].

In the absence of a human vaccine, chemotherapy remains the primary strategy for disease control. However, the therapeutic landscape is limited, and existing drugs are associated with significant drawbacks, including toxicity, high costs, and prolonged treatment regimens. These limitations are further exacerbated by the emergence of drug resistance, which threatens the sustainability of current treatment strategies [5].

The identification of new antileishmanial compounds is therefore urgent [6]. While promastigotes can be easily cultured in vitro and several assays are available to evaluate drug activity against this stage, the assessment of compound efficacy against intracellular amastigotes remains considerably more challenging. Indeed, the amastigote stage, responsible for parasite persistence within the mammalian host, represents the clinically relevant target. Currently, the gold standard method relies on microscopy-based quantification of infected cells following staining procedures, requiring manual counting of infection rates and intracellular parasite burden. However, this procedure is labour-intensive, low-throughput and highly operator-dependent, as it relies on morphological evaluation of the parasite. Reporter gene systems have become powerful tools to investigate parasite biology and to accelerate drug discovery in *Leishmania*. These approaches rely on the expression of exogenous markers that generate quantifiable signals, enabling the detection of parasite viability, gene expression, and infection dynamics in both in vitro and in vivo models [7]. In the context of drug screening, reporter-expressing parasites allow objective and quantitative assessment of parasite burden, overcoming the limitations of conventional microscopy-based methods. Fluorescent reporters, including GFP derivatives, red-shifted proteins such as mCherry or tdTomato, and near-infrared fluorescent markers, enable direct visualization of infected cells and provide spatial information on intracellular parasites across in vitro, ex vivo, and in vivo settings [8,9,10,11,12]. In addition, fluorescence-based assays allow repeated, non-destructive measurements of the same samples over time, enabling longitudinal monitoring of infection dynamics within a single experiment. In parallel, enzymatic bioluminescent reporters, such as luciferases, offer high sensitivity and a broad dynamic range, allowing precise and linear quantification of parasite load across experimental conditions [13,14,15,16]. These systems have enabled the development of high-throughput and semi-automated platforms for the identification of compounds active against *Leishmania* parasites, especially in the intramacrophage amastigote stage.

Despite these advantages, individual reporter systems present inherent limitations. Fluorescent signals can be affected by host cell autofluorescence and limited sensitivity in complex intracellular environments [17]. In contrast, bioluminescent systems, while highly sensitive, rely on the addition of exogenous substrates, and their performance can be influenced by substrate availability, stability, and cellular uptake, potentially affecting signal consistency across experimental conditions [18]. To address these challenges, dual-reporter parasites co-expressing fluorescent and bioluminescent markers have been developed as a complementary strategy [19,20,21,22]. By combining the spatial resolution of fluorescence with the high sensitivity and quantitative robustness of bioluminescence, these systems enable parallel readouts within the same parasite, allowing internal cross-validation of results and reducing artefacts associated with single-reporter assays. Nevertheless, the use of transgenic *Leishmania* strains for drug screening applications may be affected by false-positive or false-negative results, as some compounds can interfere with fluorescence- or bioluminescence-based readouts independent of their true antiparasitic activity. To overcome these limitations, we developed a dual-reporter (DR) microplate-based platform using a transgenic *L. infantum* strain stably co-expressing the red-shifted firefly luciferase PpyRE9H, fused to the tdTomato red fluorescent protein through the TY1 tag of *Saccharomyces cerevisiae* [21,23]. This system enables the evaluation of compound activity against both promastigote and intracellular amastigote stages, while providing multimodal, independent readouts from a single parasite to reduce artefacts associated with reporter-specific interference and to improve the robustness and reliability of compound screening.

## 2. Results

### 2.1. Set Up of the Microplate Assay for the Promastigote Stage of the L. infantum Transgenic Line

#### 2.1.1. Kinetics of Promastigotes Growth

**Figure 1:**
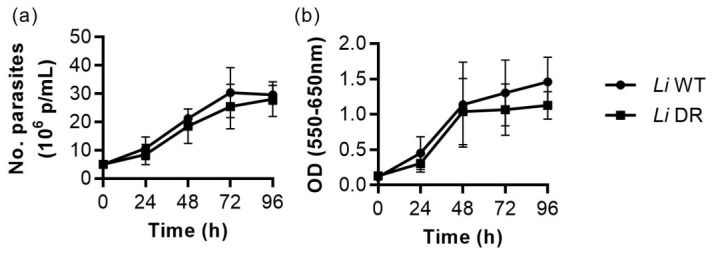
Growth kinetics of *Li* WT and *Li* DR promastigotes. Parasite growth was determined by direct counting in a Neubauer chamber (**a**) or by MTT assay measuring OD at 550–650 nm (**b**). Data are the mean ± standard deviation (SD) of at least three independent experiments performed in duplicate.

The growth of *L. infantum* wild-type (*Li* WT) and dual-reporter (*Li* DR) promastigotes was evaluated after 24, 48, 72 h, and 96 h of incubation at 23 °C using both MTT assay and direct microscopic counting. Exponential growth was observed up to 48 h by the MTT assay and to 72 h with the microscopic counting, followed by a transition to the stationary phase. The earlier plateau observed with the MTT assay may reflect a reduction in metabolic activity due to the reduction in parasite proliferation. No significant differences were observed between the two strains, indicating that the introduction of the reporter construct did not affect promastigote extracellular growth (Figure 1).

#### 2.1.2. Linearity and Sensitivity of the Luminescent, Fluorescent, and Metabolic Signals in *Li* DR Promastigotes

**Figure 2:**
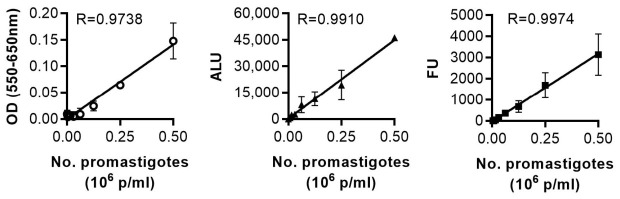
Linearity of metabolic, luminescent, and fluorescent signals in promastigotes of *Li* DR. Promastigotes were serially diluted, and absorbance (OD 550–650 nm), luminescence (ALU, arbitrary luminescence units), and fluorescence (FU, fluorescence units) were measured. Results represent the mean ± SD of three independent experiments.

Serial dilutions of promastigotes in complete RPMI medium were prepared to evaluate the linearity and sensitivity of metabolic, luminescent and fluorescent readouts. Strong linear correlations between parasite number and signal intensity were observed for all detection methods, with R values above 0.97 (Figure 2).

It is known that cell culture medium components can interfere with fluorescent readouts. Set-up experiments were therefore performed to compare results obtained using RPMI-based culture medium versus PBS. No differences in signal linearity were observed under these conditions (Figure S1), indicating that medium composition did not significantly affect fluorescence measurements.

Sensitivity was determined by calculating the signal-to-background (S/B) ratio across decreasing numbers of *Li* DR promastigotes. As shown in Table 1, the luminescent assay exhibited the highest sensitivity, reaching an S/B ratio > 4 with as few as 7.8 × 10^3^ parasites.

#### 2.1.3. Promastigote Drug Screening Assay

As an initial validation step, we confirmed that transgenesis did not affect promastigote susceptibility to reference antileishmanial drugs. The sensitivity of *Li* DR to amphotericin B, miltefosine, and paromomycin was evaluated using the standard MTT assay. Results in Table 2 show that IC_50_ values obtained with *Li* DR are comparable to those obtained with *Li* WT parasites.

**Figure 3:**
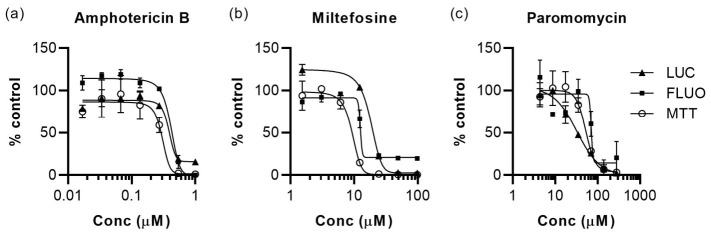
Dose–response curves of amphotericin B (**a**), miltefosine (**b**), and paromomycin (**c**) in *Li* DR promastigotes obtained using three readouts (luminescence, fluorescence, and MTT assay).

Once promastigote drug susceptibility was established, IC_50_ values were then determined using luminescent and fluorescent readouts. Drug activities measured by fluorescence, luminescence, and the MTT assay were overall consistent, with the exception of paromomycin, for which higher IC_50_ values were observed using the fluorescence-based readout (Table 2). Representative dose–response curves are shown in Figure 3.

After drug treatment, promastigotes growth was first analyzed using fluorescence. Subsequently, luciferin substrate was added, and luminescence was recorded after 10 min of incubation. For the dual-reporter measurements, the same white plate was used for both readouts. Raw fluorescence and luminescence data obtained using black vs. white plates are reported in the Supplementary Section (Table S1).

Collectively, these results indicate that transgenesis does not affect promastigote growth or susceptibility to antileishmanial drugs, and that drug activity can be reliably assessed using any of the tested readout methods. Nevertheless, direct microscopic examination of intracellular amastigotes is an important orthogonal validation approach to confirm that changes in bioluminescent and fluorescent signals reflect true antiparasitic activity. This is particularly important to exclude potential interference of tested compounds with reporter readouts.

### 2.2. Set Up of the Microplate Assay for the Intramacrophage Stage of the L. infantum Transgenic Line

#### 2.2.1. Comparison of Infectivity Between *Li* DR and *Li* WT Using the Reference Giemsa Method

The intracellular amastigote model was established using THP-1 cells differentiated into macrophages through phorbol ester treatment. The infectivity of the newly generated *Li* DR strain was evaluated by Giemsa staining and microscopic observation and compared with that of *Li* WT parasites. The percentage of infected macrophages was 20.4 ± 6.8 (range 13.8–30.61) for *Li* WT and 26.5 ± 9.7 (range 16.0–39.8) for *Li* DR. No significant differences were observed between the two strains (*p* > 0.05, *t*-test), indicating that *Li* DR exhibits infectivity levels comparable to those of *Li* WT.

**Figure 4:**
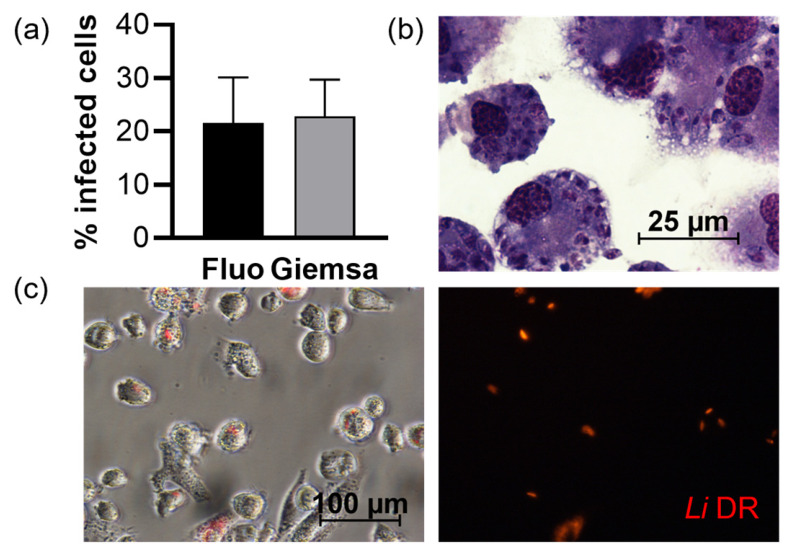
Comparison of macrophage infection by *Li* DR assessed by Giemsa staining and fluorescence microscopy. (**a**) Quantification of the percentage of infected macrophages determined by Giemsa staining and brightfield microscopy (Giemsa) or by fluorescence microscopy (Fluo). (**b**) Representative image of infected macrophages stained with Giemsa (1000× magnification, TiS Nikon). (**c**) Representative fluorescence microscopy image of infected cells (200× magnification).

When infectivity of *Li* DR was evaluated by fluorescence microscopy, the percentage of infected macrophages was similar to that obtained using Giemsa staining and brightfield microscopy (Figure 4a). Representative images of infected macrophages stained with Giemsa or observed by fluorescence microscopy are shown in Figure 4b and Figure 4c, respectively.

#### 2.2.2. Linearity and Sensitivity of the Luminescent and Fluorescent Signals in *Li* DR Intracellular Amastigotes

To assess the impact of culture medium on signal detection, measurements were first compared in complete RPMI medium vs. PBS. While luminescent signals were not influenced by medium components, fluorescence signals were reduced in complete RPMI medium compared with PBS (Table S2). Based on these results, all subsequent fluorescence and luminescence measurements were performed in PBS.

**Figure 5:**
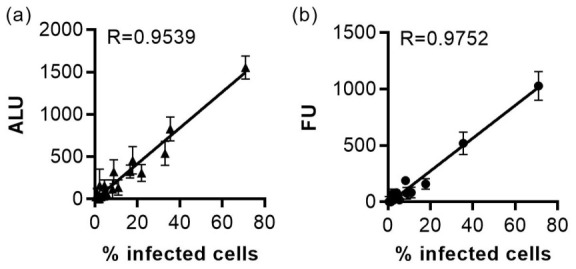
Linearity of luminescent and fluorescent signals in macrophages infected with *Li* DR amastigotes. THP-1 cells infected with *Li* DR were serially diluted with uninfected cells. (**a**) Luminescence (ALU, arbitrary luminescence units) and (**b**) fluorescence (FU, fluorescence units) were measured. Results are the mean ± SD of three independent experiments.

To evaluate signal linearity, differentiated THP-1 macrophages (dTHP-1) infected with *Li* DR (initial infection rate of approximately 70%) were serially diluted with uninfected cells, as described in the Material and Methods (Section 4.7). Results in Figure 5 show that both luminescent and fluorescent signals increased proportionally with the percentage of infected cells, demonstrating a linear relationship between infection level and signal intensity.

Sensitivity was evaluated by calculating the signal-to-background (S/B) ratio at decreasing percentages of infected dTHP-1 cells. As shown in Table 3, luminescent and fluorescent readouts exhibited comparable sensitivity, with S/B ratios greater than 2 at infection levels of as low as 3%.

#### 2.2.3. Intramacrophage Amastigote Drug-Screening Assay

Following validation of assay linearity and sensitivity, we next evaluated the suitability of the *Li* DR strain for intracellular drug screening applications. As an initial step, we assessed whether *Li* DR amastigotes exhibited drug sensitivity comparable to that of *Li* WT parasites using the standard Giemsa staining method and microscopic quantification of infected macrophages. The sensitivity of *Li* DR intracellular amastigotes to amphotericin B, miltefosine, and paromomycin was determined, and IC_50_ values were compared with those obtained for *Li* WT parasites. Results in Table 4 show that IC_50_ values obtained with *Li* DR and *Li* WT are comparable, indicating that the introduction of the dual-reporter construct did not affect drug susceptibility at the intracellular stage.

**Figure 6:**
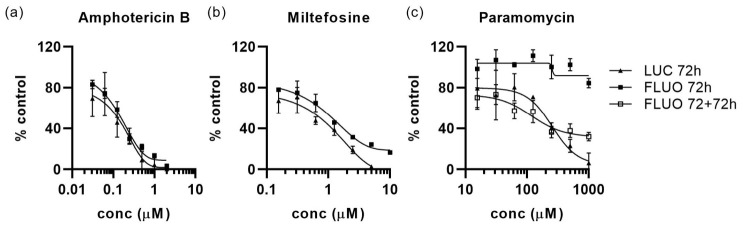
Dose–response curves of amphotericin B (**a**), miltefosine (**b**), and paromomycin (**c**) in *Li* DR intracellular amastigotes obtained using luminescent and fluorescent readouts. For paromomycin, the assay was extended for an additional 72 h (72 h + 72 h) to assess the impact of prolonged incubation on fluorescence-based measurements.

Then, IC_50_ values were calculated using luminescent and fluorescent readouts in a 96-well format. Amphotericin B and miltefosine activities were comparable among the three methods tested (Table 4). In contrast, higher IC_50_ values were obtained for paromomycin using the fluorescence-based readout compared with Giemsa and luminescence measurements. To investigate whether this discrepancy was related to the persistence of fluorescence signal in non-viable amastigotes, the assay was extended for an additional 72 h (72 + 72 h). Under these conditions, the IC_50_ value obtained by fluorescence (79.4 µM) became comparable to those obtained with the other methods. Representative dose–response curves are shown in Figure 6.

## 3. Discussion

In this study, we developed and validated an in vitro assay to evaluate antileishmanial drug activity using a transgenic *Leishmania infantum* strain (*Li* DR) stably expressing both luminescent and fluorescent reporters.

Transgenic parasites expressing reporter proteins are valuable tools for the discovery of new antiparasitic agents [24]. To this end, we generated a dual-reporter *L. infantum* strain co-expressing the red-shifted luciferase PpyRE9H fused to the red fluorescent protein tdTomato. The markers tdTomato and PpyRE9H were selected based on their previous validation in trypanosomatid imaging and their emissions at longer wavelengths than conventional GFP-based reporters and firefly luciferases. These properties reduce tissue absorption and autofluorescence, thereby enhancing signal detection in complex biological samples and improving the suitability of these reporters for ex vivo and in vivo imaging applications [25,26,27]. This genetically modified parasite line was subsequently characterized and used for the development of screening assays targeting both promastigote and intracellular amastigote stages. The assays described here provide rapid, simple, and sensitive methods for quantifying parasite growth and assessing drug activity. Parasite burden can be determined by measuring either luciferase activity (luminescence) or fluorescence.

For the promastigote stage, it was first demonstrated that *Li* DR promastigotes display growth kinetics comparable to those of the wild-type (*Li* WT) strain, characterized by an exponential phase of up to 48 h, followed by transition to the stationary phase. This indicates that the introduction of the reporter construct does not impair promastigote proliferation, an essential prerequisite for its application in drug screening assays. Accordingly, *Li* DR parasites exhibited drug susceptibility profiles similar to those of *Li* WT when tested with reference antileishmanial compounds. In chemosensitivity assays, the results obtained using fluorescence- or bioluminescence-based readouts were comparable to those obtained with the reference MTT assay, indicating that all methods can be reliably used to assess parasite viability. In addition, both luminescence and fluorescence assays were more sensitive than MTT, allowing reliable measurements at lower parasite numbers. Notably, the luminescence assay displayed greater sensitivity than fluorescence, enabling the detection of even smaller parasite numbers. This enhanced sensitivity represents a significant advantage for drug screening applications, particularly for miniaturization in a 384-well format.

Although promastigote assays are simple to perform, they have limited biological relevance, since the clinically relevant stage of *Leishmania* is the intracellular amastigote. The gold standard for determining drug sensitivity at this stage relies on microscopic quantification of intracellular parasites following staining procedures [28]. However, this approach is time-consuming, labour-intensive, and operator-dependent. The availability of a sensitive and robust assay compatible with plate-reader detection would significantly reduce analysis time and enable the development of high-throughput screening applications.

The dual-reporter *Li* DR parasites engineered in this study retained infectivity levels comparable to those of the wild-type strain, supporting the validity of this model for intracellular amastigote chemosensitivity assays. Both luminescent and fluorescent readouts exhibited good linearity and comparable sensitivity, enabling detection across a wide range of infection levels, including low percentages of infected cells. Amphotericin B and miltefosine showed consistent activity across all detection methods, confirming the reliability of the assay. In contrast, the detection of paromomycin activity through the fluorescence-based assay required an additional 72 h of incubation.

Luciferase reporters have a relatively short half-life compared with more stable fluorescent reporters such as tdTomato. Therefore, its use minimizes artefacts associated with the persistence of reporter signals in dying cells. In our assays, *Leishmania* parasites treated with paromomycin appeared viable at 72 h post-treatment when viability was assessed by fluorescence, but not when measured by luminescence. This discrepancy is likely due to the persistence of the fluorescent signal despite parasite death. Consistent with this interpretation, a clear loss of fluorescence was observed after an additional 72 h of incubation. A modest discrepancy was observed in promastigotes, where fluorescence-based IC_50_ values at 72 h were slightly higher than those obtained with the other methods. Overall, these differences likely reflect the distinct metabolic states of the two parasite stages, with the more quiescent amastigotes favouring the persistence of residual fluorescence in non-viable cells. A similar stage-dependent effect has been reported in *Plasmodium falciparum*, where pLDH assay is reliable in asexual stages but require extended incubation for the more metabolically quiescent gametocyte stage [29]. These considerations highlight the advantage of luciferase-based readouts for a more rapid detection of parasite killing. Moreover, the assay is performed using an ATP-free D-luciferin formulation, so that the bioluminescent signal relies exclusively on the metabolic activity of viable parasites. Parasites must produce both luciferase and endogenous ATP, thereby providing a physiologically relevant and robust measure of parasite viability [30].

However, a key advantage of using fluorescent *Leishmania* parasites is the possibility of repeatedly detecting the signal from the same plate at different time points, allowing the analysis of signal progression over time. Some drugs require variable time to cross cellular membranes and exert their activity, particularly against the amastigote stage. It may also be useful to monitor signal dynamics throughout the entire growth curve of the promastigote form. Moreover, fluorescence enables the direct visualization of intracellular parasites within macrophages by fluorescence microscopy, without the need for cell fixation or staining. This approach preserves cellular integrity and allows kinetic evaluation of drug effects on parasite burden at multiple post-treatment time points. Moreover, fluorescence allows the determination of the number of amastigotes per cell in a faster way compared to Giemsa staining. The presence of the fluorescent marker makes this assay suitable for high-content screening applications [31].

It is known that screening assays are prone to artefacts and signal interference arising from test compounds or other sources, including culture medium components. In the present study, only reference compounds with established antileishmanial activitywere used for the assay set up, and no newly synthesized compounds were evaluated; therefore, compound-specific interference with the fluorescence or luminescent readout cannot be excluded. To minimize interference from culture medium constituents, fluorescence and luminescence measurements in the amastigote assay were performed after replacing the culture medium with PBS. Nevertheless, test compounds themselves may substantially affect assay readouts. In particular, chemical compounds can interfere with fluorescence- or luminescence-based detection through multiple mechanisms, including autofluorescence, fluorescence quenching, light absorption, or scattering, as well as the presence of coloured, insoluble, or quenching agents [32,33]. Such interferences may lead to artifactual bioactivity signals, resulting in false-positive or false-negative outcomes, or may mask real antiparasitic activity.

A major strength of the proposed platform is the integration of multiple, orthogonal readouts, which directly addresses the challenge of assay-specific interference. Specifically, we propose the combined use of MTT, fluorescence, and bioluminescence assays for promastigote screening, and fluorescence- and bioluminescence-based assays for intracellular amastigotes. Because each detection method relies on distinct biological and physicochemical principles, interference affecting a single readout is unlikely to produce concordant effects across all three endpoints. The integration of orthogonal detection methods increases assay robustness and allows the identification of potential assay-specific interferences associated with individual compound properties.

The apparent decrease in fluorescence and luminescence observed in amastigotes may reflect their different metabolic state compared to promastigotes. Indeed, amastigotes are characterized by a low-proliferative, quiescent-like phenotype with reduced biosynthetic activity and decreased metabolic fluxes [34,35]. However, despite this reduced metabolic activity, the signal remains linear and sufficiently sensitive, supporting the use of amastigotes in screening assays.

The application of this bioluminescent/fluorescent assay is validated by its agreement with results obtained using conventional methods, namely the MTT assay for promastigotes and Giemsa staining for amastigotes. The possibility of performing sequential fluorescence and luminescence measurements in the same plate represents an additional advantage, reducing variability and increasing throughput. The reduced number of procedural steps and the overall simplicity of the method make it suitable for automated testing and support its potential use in the large-scale screening of chemical compounds. One possible limitation of this approach is the requirement of specialized equipment, including fluorescence/luminescence microplate readers and fluorescence microscopy systems, for signal detection. This may restrict the applicability of the assay in laboratories with limited access to such instrumentation. Nevertheless, these technologies are increasingly accessible in research facilities and provide advantages in sensitivity and automation.

In conclusion, this bioluminescence/fluorescence-based approach represents a sensitive, rapid, and reproducible method, providing a reliable platform for drug screening across different *Leishmania* life cycle stages.

## 4. Materials and Methods

### 4.1. Generation of Dual-Reporter Chimeric L. infantum Parasites

A 3.1 kb DNA sequence encoding the chimeric multiplex reporter protein PpyRE9H-Ty1-tdTomato was genetically engineered. The resulting cytoplasmic reporter consists of three distinct markers: the red-shifted luciferase PpyRE9H, the red fluorescent protein tdTomato, and a TY1 epitope tag. To generate dually bioluminescent and fluorescent *L. infantum* parasites, a 1.68 kb optimized version of the *Photinus pyralis* luciferase gene [36] fused to a 30 bp TY1-tag sequence [23], together with the 1.4 kb coding region of tdTomato, was amplified by PCR from the pTSARib-PpyRE9H-Ty1-tdTomato vector [21]. Amplification was performed using the primers gaAGATCTATGGAGGACGCCAAGAACATC (forward) and ataagaatGCGGCCGCTTACTTGTACAGCTCGTCCATGC (reverse), introducing BglII and NotI restriction sites, respectively. The PCR product was digested with BglII and NotI and ligated into the corresponding sites of the pLEXSY-hyg2 expression vector (Jena Bioscience GmbH, Jena, Germany). The resulting 12.25 kb pLEXSY-PpyRE9H-TY1-tdTomato construct, carrying a hygromycin B resistance cassette, was linearized with SwaI to target integration into the rDNA promoter locus. Parasites expressing the chimeric reporter protein were obtained by electroporation of *L. infantum* IPT1 promastigotes (*Li* WT) with the linearized SwaI fragment. Transfectants were selected by plating on semisolid medium containing 200 µg/mL hygromycin B (InvivoGen, Toulouse, France), allowing the isolation of individual clones, which were subsequently expanded in a liquid medium under antibiotic selection. This transgenic strain is hereafter referred to as *Li* DR (*L. infantum* Dual-Reporter).

### 4.2. Leishmania infantum and Cell Culture

The promastigote stage of *L. infantum* (MHOM/TN/80/IPT1) (*Li* WT) and of the transgenic strain *Li* DR were cultured at 23 °C in Schneider’s Drosophila Medium (Merck, Darmstadt, Germany), supplemented with 10% heat-inactivated fetal bovine serum (FBS, Euroclone SpA, Pero, Italy), 20 mM Hepes, and 2 mM L-glutamine. For all the experiments, the medium used was RPMI-1640 (Euroclone SpA, Pero, Italy) supplemented with 10% FBS, 20 mM Hepes (Euroclone SpA, Pero, Italy), and 2 mM L-glutamine (Euroclone SpA, Pero, Italy) (experiment medium).

Human acute monocytic leukemia cells (THP-1, TIB-202, ATCC, Manassas, VA, USA) were maintained at 37 °C in 5% CO_2_ in RPMI-1640 medium supplemented with 10% FBS, 50 µM 2-mercaptoethanol, 20 mM HEPES, 2 mM L-glutamine, and 10 µM sodium pyruvate (Euroclone SpA, Pero, Italy).

### 4.3. Growth Kinetics of Wild-Type and Transgenic Leishmania infantum Promastigotes

Promastigotes of the *Li* WT and *Li* DR strains were plated at a concentration of 5 × 10^5^ parasites/well in 200 μL of experimental medium and incubated at 23 °C. Parasite growth was monitored at 0, 24, 48, and 72 h using parasite counting and MTT assay. Independent wells were used at each time point for either parasite counting or the MTT assay. Three wells containing medium only were included as blanks. Parasite count was performed using a Neubauer chamber and via diluting parasites in PBS with 1% formaldehyde to avoid parasite movement. MTT assay was performed as described in Section 4.4.2.

### 4.4. Set Up of Luminescent and Fluorescent Assays in Transgenic Leishmania infantum Promastigotes

#### 4.4.1. Evaluation of Linearity and Sensitivity of the Luminescent and Fluorescent Signals

Promastigotes were plated in 96-well plates and serial 1:2 dilutions were performed in an experimental medium. The highest parasite density was 5 × 10^5^ parasites/well in 200 µL. Viability was determined using the standard MTT assay and fluorescence- or luminescence-based readout, as described below.

For each detection method, the signal-to-background (S/B) ratio was calculated using the mean optical density (OD) values from the MTT assay, fluorescence units (FU), or absolute luminescence units (ALU) measured in promastigotes (signal), and the corresponding OD, FU, or ALU values obtained from wells containing medium alone (background).

#### 4.4.2. MTT Assay on Promastigotes

Promastigote viability was evaluated by adding 20 μL of 3-(4,5-dimethylthiazol-2-yl)-2,5-diphenyltetrazolium bromide (MTT) solution (5 mg/mL in PBS) to the parasites for 3 h at 23 °C in the dark. Plates were centrifuged for 10 min at 300× *g*. The supernatants were then discarded and the dark blue formazan crystals dissolved using 100 μL of lysis buffer containing 20% (wt/vol) sodium dodecylsulfate (Merck, Darmstadt, Germany) and 40% N,N-dimethylformamide (pH 4.7 in 80% acetic acid) (Merck, Darmstadt, Germany). The plates were then read using a Synergy 4 (Agilent Biotek®, Santa Clara, CA, USA) microplate reader at a test wavelength of 550 nm and at a reference wavelength of 650 nm (OD 550–650).

#### 4.4.3. Fluorescence Assay on Promastigotes

Promastigote viability was evaluated in 96-well black plates by measuring fluorescence signals. Briefly, 100 μL of parasite suspension (from a total volume of 200 μL) was transferred to 96-well black plates. Fluorescence was measured using a Synergy 4 microplate reader with an excitation wavelength of 554 nm and an emission wavelength of 581 nm, and results were expressed as fluorescence units (FU).

#### 4.4.4. Luminescence Assay on Promastigotes

For the bioluminescence assay, promastigotes (100 μL out of a total volume of 200 μL) were transferred to 96-well white microplates, and 100 μL of the non-lysing substrate D-luciferin (1 mM in 0.1 M citrate buffer, pH 5.5) was added. Bioluminescence emission was recorded using a Synergy 4 microplate reader with an integration time of 500 ms.

### 4.5. Drug Treatment of Promastigotes

Drugs were dissolved at 10 mg/mL in DMSO (amphotericin B) or in the culture medium (Miltefosine and paromomycin), and then diluted in the experimental medium to obtain the required concentrations (final DMSO concentration lower than 1%, which is not toxic to parasites). Drugs were placed in 96-well plates, and serial dilutions were made in a final volume of 100 ul/well. Promastigotes (5 × 10^5^/100 µL/well) were distributed into the plates and incubated at 23 °C for 72 h. Parasite viability was evaluated by MTT, fluorescence, or luminescent assay, as described above.

### 4.6. Multiple Readout Assay on Promastigotes

To maximize information obtained from each experiment, IC_50_ were determined with the three different readouts from the same experimental plate. Ideally, luminescence and fluorescence measurements should be performed in separate plates, using white plates for luminescence signal and black plates for fluorescence measurements. MTT could be subsequently performed on the same sample volume used for fluorescent reading. However, to enable the simultaneous acquisition of all three readouts from a single experimental plate, promastigotes were resuspended, and 100 µL was transferred into a 96-well white plate for fluorescent measurement, followed by the addition of 100 µL of luciferin for luminescent reading after 15 min.

On the remaining promastigotes in the original experimental plate, 20 µL of MTT was added to perform the MTT assay.

### 4.7. Set Up of Luminescent and Fluorescent Assay on Intracellular Amastigotes

#### 4.7.1. Evaluation of Linearity and Sensitivity of the Luminescent/Fluorescent Signal

THP-1 cells (1.5 × 10^6^ cells/well) were plated in 6-well plates and treated with 0.1 µM of PMA for 72 h to achieve differentiation into macrophages (dTHP-1) [37]. Medium containing PMA was then removed, and cells were incubated with medium alone (control uninfected cells) or infected with stationary phase *Li* DR promastigotes at a macrophage:promastigote ratio of 1:10. After 24 h, cell monolayers were extensively washed with RPMI-1640 to remove all the non-internalized promastigotes. Cells were then detached with trypsin and counted. The percentage of infected cells was evaluated in Giemsa-stained smears, as described below. Infected cells, previously detached with trypsin, were plated at 5 × 10^4^ cells/well in white or black 96-well plates and serially diluted with uninfected cells to obtain different percentages of infected macrophages. After 24 h to achieve cell attachment, plates were analyzed for luminescent and fluorescent signals.

Fluorescence was evaluated by using a Synergy 4 microplate reader with an excitation wavelength of 554 nm and an emission wavelength of 581 nm (FU). After the first reading of the plate, the medium was removed and substituted with PBS. A second reading was performed to compare the readout obtained in either medium or PBS.

For luminescence reading, the non-lysing substrate 1 mM D-luciferin in 0.1 M citrate buffer at pH 5.5 (100 μL) was added. Luminescence emissions were acquired with 500 ms integration time using a Synergy 4 reader. Due to the low luminescent signal of intracellular amastigotes, the reading was repeated three times to compensate data variability due to luminescent signal instability.

The signal-to-background ratio (S/B) was calculated using the mean of FU (fluorescence) or the ALU (luminescence) of infected cells (signal) and FU or ALU of the uninfected cells (background).

#### 4.7.2. Giemsa Standard Assay on Intracellular Amastigote

THP-1 cells (5 × 10^4^ cells/well) were seeded in 16-chamber Lab-Tek culture slides (Merck, Darmstadt, Germany) and treated with 0.1 µM of PMA for 72 h to achieve differentiation into macrophages [37]. Medium containing PMA was then removed, and cells were infected with stationary phase *Li* DR promastigotes at a macrophage:promastigote ratio of 1:10. After 24 h, cell monolayers were washed with RPMI-1640 to remove non-internalized promastigotes. Infected cells were then incubated in the presence of drugs for 72 h. Cells were then fixed with 100% methanol and stained with Giemsa. The percentage of infected macrophages in treated and non-treated wells was determined by counting 200–300 macrophages/well under a light microscope (1000× magnification). About 10 random microscopic fields/well were observed. The infection rate in untreated control wells was defined as 100%, and the relative percentage of infection in treated samples was calculated accordingly. Dose–response curves were generated by plotting the percentage of infection versus the logarithm of compound concentration. IC_50_ values were calculated using the GraphPad Prism 8.0.2 software. Data are the mean and standard deviation of individual experiments performed in triplicate.

#### 4.7.3. Dual Fluorescent/Luminescent Assay on Intracellular Amastigotes

THP-1 cells (5 × 10^4^ cells/well) were seeded in 96-well plates and treated with 0.1 µM of PMA for 72 h to achieve differentiation into macrophages [37]. Medium with PMA was then removed and cells were infected with stationary phase *Li* DR promastigotes at a macrophage:promastigote ratio of 1:10. After 24 h, cell monolayers were extensively washed with RPMI-1640 to remove all the non-internalized promastigotes. This washing step is critical to ensure that subsequent readouts specifically reflect intracellular parasite survival, preventing potential signal interference or overestimation of parasite burden caused by residual extracellular promastigotes. Plates were then incubated in the presence of test compounds for 72 h.

Then, 96-well plates then analyzed for luminescent/fluorescent signal as described above. IC_50_ was calculated using the Gene5.10 software provided with the Synergy4 plate reader. The percent viability compared to untreated controls was plotted as a function of drug concentrations, and the curve fitting was obtained by nonlinear regression analysis using a four-parameter logistic method. The IC_50_ was extrapolated as the dose that induced a 50% inhibition of amastigote viability.

To maximize information, IC_50_ obtained with the two detection methods were determined from the same experimental plate. THP-1 cells were seeded and infected in a white 96-well plat with a clear bottom, to evaluate cell morphology and viability under an inverted light microscope. After 72 h of incubation with drugs, the medium was replaced with PBS and fluorescence was measured. Subsequently, 100 µL of D-luciferin was added and, after 15 min of incubation, luminescence was measured. Black plates were not suitable for this assay since the luminescent signal generated by intracellular amastigotes is low and requires signal enhancement provided by the white plates.

#### 4.7.4. Statistical Analysis

Differences between the IC_50_ values of antileishmanial compounds on both promastigotes and intracellular amastigotes of *Li* DR were analyzed by one-way ANOVA and Tukey’s post hoc test (GraphPad Prism 8.0.2 software).

## Figures and Tables

**Table 1:** Signal-to-background (S/B) ratios at different promastigote densities.

	Signal-to-Background (S/B) Ratios		
Number of Promastigotes × 10^3^	MTT (OD)	Fluorescence (FU)	Luminescence (ALU)
1000	8.1 ± 3.0	32.8 ± 12.0	300.5 ± 74.4
500	5.4 ± 2.2	15.5 ± 4.6	191.5 ± 67.1
250	3.2 ± 0.9	8.2 ± 3.1	108.8 ± 37.9
125	2.1 ± 0.4	3.4 ± 0.9	55.7 ± 20.8
62.5	1.5 ± 0.4	2.9 ± 0.7	33.6 ± 11.0
31.3	1.2 ± 0.5	2.2 ± 0.6	15.8 ± 3.6
15.6	1.2 ± 0.6	1.6 ± 0.4	7.8 ± 2.4
7.8	1.1 ± 0.3	1.5 ± 0.4	4.1 ± 1.0
3.9	1.2 ± 0.7	1.4 ± 0.3	2.8 ± 0.3
1.9	1.3 ± 0.8	1.3 ± 0.2	1.4 ± 0.4

**Table 2:** Activity of reference antileishmanial drugs against *Li* WT and *Li* DR promastigotes after 72 h of exposure.

	*Li* WT IC_50_ (µM)	*Li* DR IC_50_ (µM)		
	MTT Assay	MTT Assay	FLUO	LUC
Amphotericin B	0.273 ± 0.129	0.310 ± 0.152	0.224 ± 0.117	0.387 ± 0.232
Miltefosine	10.0 ± 1.7	7.5 ± 1.3	12.6 ± 1.3	15.7 ± 6.0
Paromomycin	58.8 ± 5.5	67.7 ± 19.0	115.0 ± 42.6	52.8 ± 17.1

FLUO = fluorescent assay; LUC = luminescent assay. Data are the mean ± SD of three independent experiments. Differences among IC_50_ obtained on *Li* DR using the three methods were not statistically different (*p* > 0.05, ANOVA).

**Table 3:** Signal-to-background (S/B) ratios at different percentages of infected macrophages.

	Signal-to-Background (S/B) Ratios	
% Infected Macrophages	Fluorescence (FU)	Luminescence (ALU)
30	10.2 ± 2.2	13.0 ± 2.8
15	5.9 ± 0.9	7.5 ± 1.1
7.5	3.6 ± 1.2	4.3 ± 1.4
3.25	2.1 ± 0.5	2.7 ± 0.7

**Table 4:** Activity of reference antileishmanial drugs against *Li* WT or *Li* DR intracellular amastigotes after 72 h of exposure.

	*Li* WT IC_50_ (µM)	*Li* DR IC_50_ (µM)		
	Giemsa	Giemsa	FLUO	LUC
Amphotericin B	0.187 ± 0.031	0.459 ± 0.311	0.267 ± 0.178	0.187 ± 0.090
Miltefosine	0.712 ± 0.263	1.123 ± 0.323	0.974 ± 0.127	0.784 ± 0.180
Paromomycin	115.8 ± 39.1	437.2 ± 171.3	>1000	176.3 ± 39.8

FLUO = fluorescent assay; LUC = luminescent assay. Data are the mean ± SD of at least three independent experiments. Differences among IC_50_ obtained on *Li* DR with the Giemsa staining and luminescent readouts were not significantly different (*p* < 0.05, ANOVA).

## Data Availability

Data is contained within the article or Supplementary Material.

## Supplementary Materials

The following supporting information can be downloaded at: https://www.mdpi.com/article/10.3390/molecules31122041/s1, Figure S1: Comparison between the fluorescent signal of promastigotes in RPMI-based culture medium or PBS; Table S1: Comparison between the fluorescent and luminescent signal from control (promastigotes) and background (medium only) samples in black vs. white plates; Table S2: Comparison between the fluorescent and luminescent signal from control (infected cells) and background (uninfected cells) samples in medium vs. PBS.

## Abbreviations

The following abbreviations are used in this manuscript:

*Li* WT	*L. infantum* Wild-Type
*Li* DR	*L. infantum* Dual-Reporter
S/B	Signal-to-Background
ALU	Arbitrary Luminescence Units
FU	Fluorescence Units
