# Supplementary material for: A Dual-Reporter Fluorescence–Luminescence Assay for Drug Screening in Promastigote and Intracellular Amastigote Stages of Leishmania
Source: Molecules. 2026 Jun 11;31(12):2041. doi: 10.3390/molecules31122041 (PMC13305902; doi:10.3390/molecules31122041)
Supplement: Supplementary file 1 [file molecules-31-02041-s001.zip › molecules-4322770-supplementary.pdf]

A Dual-Reporter Fluorescence–Luminescence Assay for Drug Screening in Promastigote and Intracellular Amastigote Stages of *Leishmania*

Sarah D'Alessandro[1], Silvia Parapini[2]*, Estefanía Calvo Alvarez[1], Federica Perego[3], Gaia Mazza[3], Abirla Murugan[3], Nicoletta Basilico[3]

[1] Department of Pharmacological and Biomolecular Sciences, University of Milan, 20133 Milan, Italy.

[2] Department of Biomedical Sciences for Health, University of Milan, 20133 Milan, Italy.

[3] Department of Biomedical, Surgical and Dental Sciences, University of Milan, 20122 Milan, Italy.

*Corresponding author

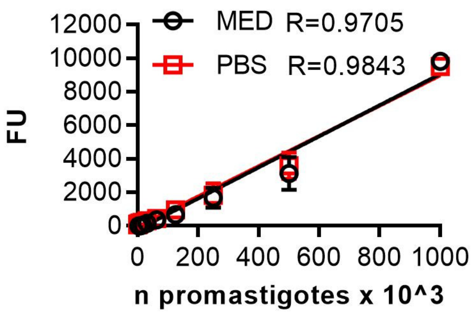

Figure S1. Comparison between the fluorescent signal of promastigotes in RPMI-based culture medium or PBS

Table S1. Comparison between the fluorescent and luminescent signal from control (promastigotes) and background (medium only) samples in black vs white plates

		Control	Background
**Fluorescence (FU)**	Black plate	1684±903	527±31
	White plate	6583±874	302±91
**Luminescence (ALU)**	Black plate	5962±2044	17±4
	White plate	272168±99166	961±169

Table S2. Comparison between the fluorescent and luminescent signal from control (infected cells) and background (uninfected cells) samples in medium vs PBS

		Control	Background
**Fluorescence (FU)**	Medium	14711±11108	6394±418
	PBS	42203±4141	7660±274
**Luminescence (ALU)**	Medium	407±228	49±16
	PBS	573±279	67±15

FU= Fluorescence units; ALU= Arbitrary Luminescence Units. Data are the mean ± sd of at least four independent experiments
